# Three-Dimensional Models as a New Frontier for Studying the Role of Proteoglycans in the Normal and Malignant Breast Microenvironment

**DOI:** 10.3389/fcell.2020.569454

**Published:** 2020-10-09

**Authors:** Julien Clegg, Maria K. Koch, Erik W. Thompson, Larisa M. Haupt, Priyakshi Kalita-de Croft, Laura J. Bray

**Affiliations:** ^1^Institute of Health and Biomedical Innovation, Queensland University of Technology, Kelvin Grove, QLD, Australia; ^2^School of Biomedical Sciences, Faculty of Health, Queensland University of Technology, Brisbane, QLD, Australia; ^3^Translational Research Institute, Woolloongabba, QLD, Australia; ^4^Centre for Genomics and Personalized Health, Genomics Research Centre, School of Biomedical Sciences, Institute of Health and Biomedical Innovation, Queensland University of Technology, Kelvin Grove, QLD, Australia; ^5^UQ Centre for Clinical Research, Faculty of Medicine, University of Queensland, Herston, QLD, Australia; ^6^Faculty of Science and Engineering, School of Mechanical, Medical and Process Engineering, Queensland University of Technology, Brisbane, QLD, Australia

**Keywords:** breast cancer, proteoglycans (PGs), extracellular matrix (ECM), 3D models, tumor microenvironment (TME)

## Abstract

The extracellular matrix (ECM) provides cues to direct mammogenesis, tumourigenesis and metastatic processes. Over the past several decades, two-dimensional (2D) culture models have been invaluable in furthering our understanding of the tumor microenvironment (TME), however, they still do not accurately emulate the associated biological complexities. In contrast, three-dimensional (3D) culture models provide a more physiologically relevant platform to study relevant physicochemical signals, stromal-epithelial cell interactions, vascular and immune components, and cell-ECM interactions in the human breast microenvironment. A common thread that may weave these multiple interactions are the proteoglycans (PGs), a prominent family of molecules in breast tissue. This review will discuss how these PGs contribute to the breast cancer TME and provide a summary of the traditional and emerging technologies that have been utilized to better understand the role of PGs during malignant transformation. Furthermore, this review will emphasize the differences that PGs exhibit between normal tissues and tumor ECM, providing a rationale for the investigation of underexplored roles of PGs in breast cancer progression using state-of-the-art 3D culture models.

## Introduction

Proteoglycans (PGs) are functional proteins that constitute major signaling and structural components of the breast microenvironment, playing a role in morphogenesis, vascularisation, tumor progression and metastasis. During breast cancer development and growth, PG expression undergoes substantial changes within both the tumor cells and the tumor microenvironment (TME). These changes result in the alteration of cell proliferation, survival adhesion and migration, making PGs a potential target for breast cancer treatments. While PGs have been studied for some decades, the models of choice to study their function and contributions toward drug development has varied. In this review, we present an overview of PGs in the human breast microenvironment along with state-of-the-art PG-focussed investigations in mammary development and breast cancer, and the emerging role of three-dimensional (3D) culture methods in modeling PGs, followed by a discussion on novel bioengineered scaffold-based approaches for future studies. We critically discuss the benefits and limitations of current models and present emerging concepts of novel cancer model systems that can mimic the role of PGs in human breast biology and its malignant transformation.

## Breast Cancer

Globally, breast cancer is the most frequent cancer in women after nonmelanoma skin cancer. The worldwide incidence of newly diagnosed breast cancer in 2018 was estimated to be 2.1 million women, which approximates to one new case diagnosed every 18 s, and 626,679 women with breast cancer succumbing to the disease in 2018 (Bray et al., 2018). As a disease, breast cancer is molecularly heterogeneous. The seminal study by Perou and Sorlie defined an intrinsic classification of four subtypes of breast cancer (Perou et al., 2000): luminal A, luminal B, basal-like and human epidermal growth factor receptor 2 (HER2). This study initiated a shift toward more biology-based approaches in the clinical management of breast cancer. This also led to further molecular subtyping in numerous studies to provide clinically relevant subgroups (Lehmann et al., 2011; Cancer Genome Atlas Network, 2012; Nik-Zainal et al., 2016; Jackson et al., 2020).

Due to this heterogeneity, breast cancer treatment paradigms have evolved over the last decade. Despite this progress, some characteristics such as the impact of the regional tumor burden or size and patterns of metastases are shared amongst breast cancers and influence therapy. Treatment decisions are generally impacted by the clinical features (stage and grade), histological biomarkers and molecular features of the cancer. In the case of non-metastatic tumors, the main goal is to eradicate the tumor from the breast and regional lymph nodes, thus preventing metastatic spread. This includes surgical resection and sampling and/or removal of axillary lymph nodes. Furthermore, postoperative radiation is also commonly used. Systemic therapy may be performed preoperatively (neoadjuvant), postoperatively (adjuvant), or both, and it is mainly guided by breast cancer subtype, stage and grade. In clinical practice, three biomarkers are used to distinguish the subtypes and guide targeted therapies; estrogen receptor (ER), progesterone receptor (PR), and HER2. Tumors expressing ER and PR are called hormone receptor-positive, whereas tumors that do not express ER, PR, and HER2 are termed triple-negative breast cancers (TNBCs). Hormone-receptor positive breast cancers usually receive endocrine therapy such as tamoxifen or aromatase inhibitors, while patients with more advanced disease or those refractory to endocrine therapy also receive chemotherapy (Burstein et al., 2010). Tumors expressing HER2 receptor receive anti-HER2 monoclonal antibodies and/or HER2-targeted agents, along with chemotherapy (Lehmann et al., 2011). Chemotherapy remains the mainstay treatment for TNBCs, however, poly ADP-ribose polymerase inhibitors (PARPi) have emerged as a promising therapy that exploit synthetic lethality associated with defective DNA damage repair mechanisms in cancer (Dulaney et al., 2017).

## Proteoglycans and Their Role in Human Mammary Tissue

In breast tissue, normal and malignant epithelial cells interact with their surrounding microenvironment, determining both cell fate and disease progression. The microenvironment contains various cell types, including endothelial cells, endothelial precursors, pericytes, adipocytes, fibroblasts, and immune cell populations. These distinct cell types are embedded in a biofunctional extracellular matrix (ECM), which is composed of numerous molecules including PGs, collagen, laminin, fibronectin, and their associated proteases (Naba et al., 2012). PGs, which contain a core protein to which different numbers and types of glycosaminoglycan (GAG) side chains are attached, are a major ECM component and are additionally expressed on cell surfaces. The cellular location and types of GAG side chains built upon a core protein are used to classify PGs into different groups (Ruoslahti, 1988). They vary extensively in structure, localisation and functionality (Pearson et al., 1983). Based upon their localization, PGs fall into four families (Iozzo and Schaefer, 2015) (intracellular, cell-surface, pericellular, and extracellular), with the functionality of PGs occurring mainly through their GAG content. Serglycin is the sole PG characterized to be intracellular in breast tissue (Scully et al., 2012) and is widely investigated in cancer (Korpetinou et al., 2014). Pericellular and cell surface PGs include decorin, versican, glypican, and perlecan as well as collagens XVIII and XV. The extracellular PGs comprise hyaluronan-binding hyalectans, small-leucine rich PGs (SLRPs) and calcium-binding heparan sulfate PGs (HSPGs) (Nikitovic et al., 2018). This stratification is extremely useful for discussing the function of these PGs from the cellular biology perspective and it has been reviewed extensively by Nikitovic and colleagues (Nikitovic et al., 2018). From a translational and clinical perspective, PGs localized to different compartments may contribute to the same function therefore, this review will mostly focus on the subgroups defined by GAG type; the implicated functionally interacting parts of PGs. We will describe how uniquely each of these contribute to normal function and pathophysiological features at different stages of the development of the mammary gland as well its malignant transformation. These GAGs include; heparan sulfate (HS), chondroitin sulfate (CS), dermatan sulfate (DS), keratan sulfate (KS), and hyaluronic acid (HA).

## Modeling the Function of PGs in Healthy Mammary Tissue

**FIGURE 1:**
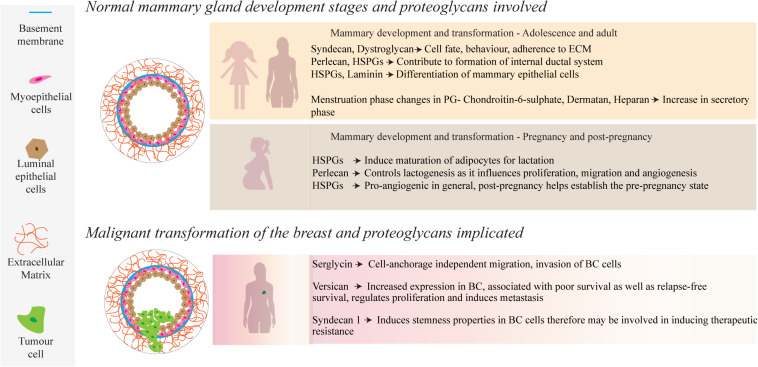
During normal mammary development, a number of PGs, such as those containing HS side-chains, interact with growth factors, ECM molecules and cell receptors to mediate physiological and cellular remodeling. Several of these PGs also play a role in mammary tumor progression through up or down regulation of their local concentrations.

Attaining an understanding of the role of PGs in mammary development and homeostatic processes is central to understanding their significance in the pathology of breast cancer. Tissue development and remodeling are seen throughout life, where the fetus undergoes initial development to hormonal directed tissue remodeling through adolescence, adulthood and the periods prior, during and following pregnancy, enabled by PGs found within the mammary ECM and cell surfaces (Baker et al., 2017; Figure 1). During fetal development, transmembrane matrix members of the syndecan PG family (syndecans 1–4), synergistically interact with integrins to aid in embryonic angiogenesis (Morgan et al., 2007). Syndecans, along with integrins, work with the glycoprotein dystroglycan to adhere to the cytoskeleton and can influence cell fate and behavior, along with other cell-ECM and cell-cell interactions (Barresi and Campbell, 2006; Morgan et al., 2007; Garner et al., 2011). Moreover, a study by Liu et al. (2003) found that syndecan-1 greatly influences tertiary ductal branching in mice, further highlighting the importance of the syndecan PG family in mammary development (Liu et al., 2003).

There is a dynamic and ever-evolving microenvironmental remodeling that occurs throughout adolescence and pregnancy, such as ductal morphogenesis driven by cell-ECM interactions with the basement membrane (Hinck and Silberstein, 2005). Epithelial proliferation and migration contribute to the development of the internal ductal system, facilitated by estrogen and local growth factors that interact with epithelial and stromal cells (Lu et al., 2008). Perlecan, a HSPG found largely in the basement membrane, captures anionic growth factors such as stromal fibroblast-growth factor 10 (FGF10), delivering it to the epithelium for growth (Patel et al., 2007). The complex interplay between such HSPG-bound growth factors is modulated by the enzyme heparanase, which cleaves HS side chains and liberates the free growth factors (Chhabra and Ferro, 2018).

Another feature during breast development is the migration and maturation of adipocytes, which comprise a large part of the mammary stroma and are involved in energy delivery for lactation (Gouon-Evans and Pollard, 2002; Alvarenga et al., 2019). Fat deposits in the mammary microenvironment, and further, their maturation and adipocyte growth play an important role in supporting homeostasis and later remodeling and milk production during and following pregnancy (Muschler and Streuli, 2010). It has been reported by Wilsie et al. (2005) that adipocyte growth is heavily dependent upon cell membrane HSPGs, along with the onset of estrogen production by pre-adipocytes (Wilsie et al., 2005). During pregnancy, hormone-driven changes are characterized by an increase in epithelial density, ductal growth and a complexifying of the regional vascularization (Macias and Hinck, 2012). This is facilitated by PGs that bind with growth factors, activating cellular pathways to upregulate cell growth and proliferation (Figure 1). As an example, the HSPG syndecan-4 has been postulated to have a functional role in lactogenesis, including lobuloalveolar development as seen by its involvement in proliferation, migration and angiogenesis (Moon et al., 2005; Bishop et al., 2007; Morrison and Cutler, 2010). The HS GAGs bind with many pro-angiogenic factors that include; transforming growth factor-beta (TGF-β), hepatocyte growth factor, heparin-binding EGF, and FGFs-1, 2, 4, 7, and 10. This coordinated network directly facilitates the controlled proliferation of the endothelium and migration of cells for angiogenesis. A lack of HS GAGs leads to a failure of lobuloalveolar development, resulting in a lack of milk production (Segev et al., 2004; Crawford et al., 2010). Apoptotic events to re-establish the pre-pregnancy adult state of the mammary microenvironment are also directly regulated by HSPGs through interactions with Fas ligand and tumor necrosis factor-related apoptosis-inducing ligand (TRAIL), among others (Liu et al., 2004; Wu et al., 2012).

**FIGURE 2:**
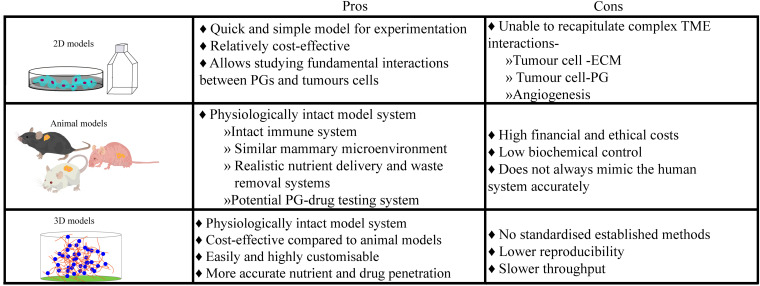
Culture models ultimately have an innate advantage over histological samples taken from breast cancer patients, as they provide a controlled but dynamic environment for experimentation over longer periods. However, each culture model system presents their own unique limitations, leading researchers to choose which system is best for their research or needing to use multiple culture systems.

Ensuring that the right model is used for the research question is imperative in all investigations (Figure 2). 2D systems, in conjunction with animal studies (Pumphrey et al., 2002; Liu et al., 2003; Kelly et al., 2005; Bischof et al., 2013) and biopsy material, have facilitated our current understanding of the biosynthesis of PGs, their biochemistry including interactions with other biological substances and their functions within the development of normal and pathological mammary microenvironments, as extensively reviewed by Theocharis (Theocharis et al., 2015). In this review, we chose to focus on the details of studies using patient biopsies or cell suspensions in a 3D context. Histological studies of human specimens have played a pivotal role in the elucidation of healthy mammary development, and the life-long remodeling that occurs. The study conducted by Ferguson et al. (1992) investigated the changes of ECM molecules during the menstrual cycle in human females (Ferguson et al., 1992). It was discovered that over the 4-week menstrual cycle, ECM molecules such as heparan and chondroitin sulfate PGs, changed in their concentration and localization. Understanding the anatomical and molecular changes of the healthy mammary microenvironment also allows for a better understanding of the contribution of PGs to the development of breast TMEs. For example, biopsy studies by De Lima et al. (2012) and Ricciardelli et al. (2002) compared normal and cancerous mammary tissue and discovered independently, that the levels of PGs in the healthy tissue were widely different when compared with pathological tissue (Ricciardelli et al., 2002; De Lima et al., 2012), including an upregulation of heparan and dermatan sulfate PGs. Moreover, a recent study using breast tissue explants cultured on a gelatin sponge investigated the role of PGs in mammographic density (Huang et al., 2020). Application of a syndecan-1 inhibitory peptide, synstatin, to denser breast tissue reduced the amount of fibrillar collagen and overall mammographic density. However, histological studies and 2D models/cultures present a number of issues, predominantly in their inability to directly observe the mechanistic pathways that ultimately drive normal development and homeostatic processes (Figure 2). Future studies could take advantage of more sophisticated 3D platforms, explored in the following sections.

## Modeling the Function of Proteoglycans in the Breast TME

Breast cancer is a dynamic heterogeneous pathophysiological disease and while we have formulated comprehensive mechanisms of development underlining the disease, we lack an equally comprehensive understanding of how the ECM contributes to the known cancer hallmarks and how we can leverage this understanding into developing novel treatments. The TME remodels the associated stroma, resulting in an increased content of various PGs (Figure 1). Serglycin promotes anchorage-independent cell migration, growth and invasion of breast cancer cells and is associated with aggressive properties of cancer (Korpetinou et al., 2013). In contrast, lumican possesses anti-migratory and anti-invasive properties as demonstrated in aggressive breast cancer cell lines (Karamanou et al., 2017). Studies by Dawoody Nejad et al. (2017) and Recktenwald et al. (2012) have investigated decorin and biglycan, respectively, using cell culture dishes (Recktenwald et al., 2012; Dawoody Nejad et al., 2017). Dawoody found that decorin and fibromodulin overexpression downregulated NF-kB and TGF-β1, vital signaling complexes during epithelial-mesenchymal transitioning, in the metastatic 4T1 breast cancer cell line, which may lead to an opportunity to prevent metastatic phenotype conversion in cancer cells. Recktenwald demonstrated that downregulation of biglycan led to the promotion of cell proliferation and migration in HER2^+^ cell lines, suggesting that biglycan also has an inhibitory effect on tumor cells.

Overall, growing evidence indicates the importance of versican as a potential PG involved in mammary carcinogenesis. Versican is a large PG that is localized to the ECM and can be found in many tissue regions (Wight, 2002). It is one of the most widely researched PGs in cancer. and has been implicated within cancerous samples but not within their healthy control samples (Ricciardelli et al., 2002). Ricciardelli et al. (2002) investigated versican, using biopsy material from 58 node-negative patients and found that versican was detected in all samples from confirmed breast cancer patients. Additionally, the study showed that the relapse rate was lower in patients with lower versican levels in their biopsy specimens and increased in samples with higher versican levels.

These studies by Dawoody Nejad et al. (2017); Recktenwald et al. (2012), and Ricciardelli et al. (2002) provide examples of specific PGs but did not explore their mechanisms of action, nor their explicit interactions with cancerous cells. Histology-based assessments such as these could be considered as static assessments, while dynamic assessments using culture models allow for more extensive investigations and facilitate examination of a changing microenvironment. Versican also plays an essential role in the structural architecture of vascular walls, and is upregulated in breast cancer (Du et al., 2010), regulating tumor growth by promoting angiogenesis (Asano et al., 2017). Furthermore, it assists in regulating proliferation and inducing metastasis through its interaction with various ECM components and tumor cells (Laoui et al., 2011; Du et al., 2013). Interestingly, dos Reis and co-workers recently demonstrated the correlation of versican expression with tumor associated macrophage (TAM) accumulation and progression using two *in vivo* models of mammary carcinomas (Dos Reis et al., 2019). The evidence from various studies highlights the unique roles that each PG plays in mammary tumourgenesis and progression. Uniform upregulation or downregulation is clearly not what propagates this progression, but rather a complex interplay of up and downregulation leading to a variety of signaling interactions, ultimately resulting in increased proliferation, invasion and poor survival outcomes (Troup et al., 2003; Prinz et al., 2011).

In *in vitro* studies, a potential role for syndecan-1 in modulating breast cancer brain metastasis has been identified (Sayyad et al., 2019). On the other hand, *in vivo* studies such as by Pumphrey et al. (2002), explored the interactions of syndecan-1 HSPG analogs that were synthetically produced by carbodiimide conjugation of GAG chains. Their anticancer properties were evaluated by injection into breast tumors that were grown in nude mice in which they observed a significant reduction in growth without cytotoxicity to neighboring healthy tissue. However, animal models do come with their limitations and have led researchers to look for alternatives. They are financially and ethically expensive and although *in vivo* models encapsulate a physiologically robust and similar microenvironment to that found in humans, they do not fully represent human biology nor are a platform for customisability of microenvironmental parameters. This is where 3D models aim to exploit the best qualities of both 2D and *in vivo* models and combine them in a cost-effective, reproducible and most importantly, physiologically relevant, yet highly customisable, human model (Figure 2). In an attempt to combat the difficulties presented by previous models, 3D culture models such as organ-on-a-chip, hydrogels, scaffolds, and spheroid formation provide viable *in vitro* alternatives that extend throughout many cell-based medical research fields. These customisable models predominantly aim to recreate a physiologically relevant ECM to better mimic biologically relevant cell-cell and cell-ECM interactions.

Naturally derived hydrogels such as Matrigel and collagen provide a growth factor rich 3D environment for cancer cells to grow in. Matrigel, a reconstituted basement membrane extract from the Engelbreth-Holm-Swarm (EHS) sarcoma (Vukicevic et al., 1992), is rich in basement membrane components, including the HSPG perlecan and has been used to study the invasion of breast cancer cells in response to syndecan-1 knockdown (Nikolova et al., 2009; Ibrahim et al., 2013). The authors found that proteolytic conversion of syndecan-1 from being membrane-bound to a soluble form switched breast cancer cells from a proliferative to an invasive phenotype (Nikolova et al., 2009). Consistent with this, the overexpression of miR-10b resulted in downregulated syndecan-1 and increased invasiveness into a Matrigel matrix (Ibrahim et al., 2013). Moreover, syndecan-1 has further been found to modulate the phenotype of cancer stem cells in triple negative breast cancer types, and when silenced, reduced 3D spheroid formation *in vitro* (Ibrahim et al., 2017). Collagen-derived hydrogels have also been used to study PGs. In a recent study by Singh et al. (2019), MCF-7 and MDA-MB-231 cells were cultured in 3D collagen hydrogels to explore the role of dermatan sulfate PGs in breast cancer invasion (Singh et al., 2019). The authors artificially decreased the expression of iduronate-2-sulphatase (IDS) in the cell lines, increasing the levels of dermatan sulfate, which led to a more mesenchymal morphology and increased breast cancer cell invasion into a collagen matrix. A recent study from Karamanou et al. (2020) also utilized collagen-derived hydrogels to explore the effects of lumican on cell adhesion, including the molecular mechanisms behind its anti-cancer activities, demonstrating its potential as a target for anti-cancer therapies (Karamanou et al., 2020).

HA-based hydrogels have also been employed to investigate GAG/PG relationships, consisting of a scaffold architecture aimed to provide a biomimetic ECM for PG signaling. Bonnesœur et al. (2020) demonstrated that HA-hydrogels were capable of supporting both mammary and brain-derived cancer cell lines while showing the tunability and biomimetic capabilities of PG-based hydrogels (Bonnesœur et al., 2020). Moreover, a study by Fisher et al. (2015) utilized HA and matrix-metalloproteinase (MMP) cleavable peptides to investigate MDA-MB-231 invasion capabilities (Fisher et al., 2015). In this case, HA was furan-modified and combined with a bismaleimide peptide crosslinker resulting in a Diels-Alder click reaction. The researchers created several hydrogels with and without MMP-cleavable peptides. Increased crosslinking density resulted in decreased invasion, while hydrogels incorporated with MMPs and low crosslinking density, showed higher levels of invasion. This was corroborated by Hubka et al. (2019), who also utilized thiolated HA with a poly(ethylene glycol)diacrylate (PEGDA) crosslinker to form hydrogels in order to investigate the role of perlecan domain 1 (Hubka et al., 2019). Using FGF-2 along with the perlecan domain 1, they demonstrated higher migratory and invasive behaviors in sections of the hydrogels with higher concentrations of perlecan domain 1 and thereby FGF-2. Baker and colleagues developed this further (formed by a Diels-Alder reaction) when they assessed drug infiltration into spheroid colonies within their HA-hydrogels in MCF-7 and T47D cells (Baker et al., 2017). They saw that not only was there reduced penetration into inner cells by the drugs, but drug and multi-drug resistance occurred, and more accurately represented the phenomenon of drug resistance that occurs in the clinic.

An example of a non-hydrogel 3D *in vitro* system was formulated by Hinderer et al. (2018), where electrospun scaffolds were functionalised to assess decorin interaction with endothelial cells (Hinderer et al., 2018). Decorin and stromal cell-derived factor-1 (SDF-1) coated scaffolds showed high levels of adhesion, migration and interaction with human endothelial progenitor cells (hEPCs) when compared to non-functionalized scaffold controls. Further, they found that decorin had higher levels of interaction and influence over the migration of the hEPCs compared to SDF-1. Le Borgne-Rochet et al. (2019), also using a scaffold system, studied the role of decorin in cell migration, illustrating the interactions decorin has with the adhesion molecule P-cadherin (Le Borgne-Rochet et al., 2019). This further supported Hindere’s work regarding the many interactions of decorin and showed that P-cadherin mediated the directional cell migration and collagen fiber orientation required the presence of decorin. They also discovered that decorin activation resulted in upregulation of β1 integrin and the β-Pix/CDC42 axis promoting directional cell migration.

## Conclusion and Perspectives

These studies implicate a multifaceted role for PGs in breast cancer. It is tempting to suggest that the stromal-breast tumor PG microenvironment may shape the fate of these programmable ever adapting tumor cells. However, it is a balancing act where subtle changes in the content of PGs and associated ECM may be utilized by the tumor cells to overcome the intrinsic resistances and hostilities presented to them. Current findings from the literature warrant undertaking comprehensive mechanistic studies where this balance could be exploited to favor a healthy microenvironment. To date, the study of breast PGs in a 3D *in vitro* context has been limited. While 2D cultures have been utilized for their ease-of-use, there is a substantial difference in cellular behavior and morphology when the context of the cellular microenvironment is changed (Weigelt et al., 2014). Advances in 3D cell culture have led to novel findings, including biological events that transpire during cancer development that were previously not known. The establishment of these relatively new systems as dominant platforms for medical research, will enable further novel findings, including further study of the complex interactions of PGs. In particular, 3D model systems provide the ideal platform to assess PGs with a significant lack of studies leveraging these physiological relevant systems currently employed in breast cancer and other cancer research.

State-of-the-art biomaterials and tissue engineering strategies enable the reconstruction of different microenvironments by providing biomechanical and biochemical compositions specific to various tissues. In previous work, we have shown how semi-synthetic hydrogels prepared from PEG and heparin GAGs represent a platform for TME modeling (Bray et al., 2015). Likewise, hydrogels formed from gelatin-methacryloyl (GelMA) have been used to successfully recapitulate breast cancer invasion and chemoresponses *in vitro* (Yue et al., 2015; Loessner et al., 2016; Donaldson et al., 2018; Meinert et al., 2018). Exploiting such biohybrid 3D matrices allows for the control of batch-to-batch physicochemical properties, which are known to affect cellular behavior in the TME. The implementation of 3D systems further allows for the extensive analysis of ECM and basement membrane deposition by the cells in their surrounding microenvironment. In order to study the role of PGs in breast cancer *in vitro*, attention should be paid to give microenvironmental and PG context, and to allow for the heterogeneous culture of various supporting cell types resulting from patient tissues (Foley, 2017). Finally, dynamic culture conditions, such as bioreactors (Selden and Fuller, 2018), microfluidics (Shang et al., 2019), and tumor-on-chip (Rothbauer et al., 2019; Sun et al., 2019) technologies, exemplify options to raise the physiological relevance of PG studies specific to the breast tissue microenvironment. Taken together, we anticipate that the development of novel and sophisticated 3D models in which to study the role of PGs in the breast microenvironment will allow for the identification of novel therapeutic targets for preventing and treating malignant transformation in breast cancer patients, such as in the case of the syndecan-1 inhibitor, synstatin (Beauvais et al., 2009; Jung et al., 2016).

## Author Contributions

LB and PK-dC delineated the topic and outline. JC, PK-dC, and LB reviewed and evaluated the literature, designed, and wrote the manuscript. MK, LH, and ET provided feedback and edited the final manuscript.

## Conflict of Interest

The authors declare that the research was conducted in the absence of any commercial or financial relationships that could be construed as a potential conflict of interest.
